# Patient Knowledge on Malaria Symptoms Is a Key to Promoting Universal Access of Patients to Effective Malaria Treatment in Palawan, the Philippines

**DOI:** 10.1371/journal.pone.0127858

**Published:** 2015-06-16

**Authors:** Emilie Louise Akiko Matsumoto-Takahashi, Pilarita Tongol-Rivera, Elena A. Villacorte, Ray U. Angluben, Masamine Jimba, Shigeyuki Kano

**Affiliations:** 1 Department of Tropical Medicine and Malaria, Research Institute, National Center for Global Health and Medicine, Tokyo, Japan; 2 Department of Community and Global Health, Graduate School of Medicine, The University of Tokyo, Tokyo, Japan; 3 Department of Parasitology, College of Public Health, University of the Philippines Manila, Manila, the Philippines; 4 Kilusan Ligtas Malaria/Pilipinas Shell Foundation, Inc., SPS Government Center, Palawan City, the Philippines; Universitat Rovira i Virgili, SPAIN

## Abstract

**Introduction:**

Palawan, where health care facilities are still limited, is one of the most malaria endemic provinces in the Philippines. Since 1999, microscopists (community health workers) have been trained in malaria diagnosis and feasibility of early diagnosis and treatments have been enhanced throughout the province. To accelerate the universal access of malaria patients to diagnostic testing in Palawan, positive health seeking behavior should be encouraged when malaria infection is suspected.

**Methods:**

In this cross-sectional study, structured interviews were carried out with residents (N = 218) of 20 remote malaria-endemic villages throughout Palawan with a history of suspected malaria from January to February in 2012. Structural equation modeling (SEM) was conducted to determine factors associated with appropriate treatment, which included: (1) socio-demographic characteristics; (2) proximity to a health facility; (3) health seeking behavior; (4) knowledge on malaria; (5) participation in community awareness-raising activities.

**Results:**

Three factors independently associated with appropriate treatment were identified by SEM (CMIN = 10.5, df = 11, CFI = 1.000, RMSEA = .000): “living near microscopist” (*p* < 0.001), “not living near private pharmacy” (*p* < 0.01), and “having severe symptoms” (*p* < 0.01). “Severe symptoms” were positively correlated with more “knowledge on malaria symptoms” (*p* < 0.001). This knowledge was significantly increased by attending “community awareness-raising activities by microscopists” (*p* < 0.001).

**Conclusions:**

In the resource-limited settings, microscopists played a significant role in providing appropriate treatment to all participants with severe malaria symptoms. However, it was considered that knowledge on malaria symptoms made participants more aware of their symptoms, and further progressed self-triage. Strengthening this recognition sensitivity and making residents aware of nearby microscopists may be the keys to accelerating universal access to effective malaria treatment in Palawan.

## Introduction

Until very recently, the residents of Palawan province, the Philippines, lacked access to timely and adequate medical care for malaria, which is one of the most serious parasitic infections in the country [1–6]. Prompt diagnosis and proper treatment are the principal strategies for controlling malaria [7]. Thus, a total of 344 volunteers (one for each endemic village, excluding 76 non-endemic villages) were trained as microscopists. Microscopists identify malaria infection and species of parasites, by microscopic examination of Giemsa-stained blood smears. Microscopists, under the supervision of midwives, also administer first-line anti-malarial drugs to malaria patients.

Microscopists in Palawan are trained as community health workers (CHWs). They specialize in malaria microscopic diagnosis and treatment. In rural areas such as Palawan, where there is a recognized paucity of formal public and private healthcare providers, the utilization of CHWs is a potentially inexpensive, effective, and sustainable approach for bringing malaria diagnosis and treatment closer to households [8–10]. Through this community-based strategy, which was launched in 1999 [11–13], the annual parasite index (API) per 1,000 population in Palawan decreased from 27.6 in 2004 to 13.0 in 2010, however, the annual number of cases in 2012 still exceeded 1,000 [2–6].

Two studies which aimed to form strategies to reduce malaria re-infection among ex-patients who visited microscopists in Palawan have been conducted [12,13]. The first suggested that enhancement of “service quality” and “ability in malaria microscopy” were the keys to strengthening community awareness-raising activities for malaria prevention by microscopists [12]. The second study suggested that these activities should be effective and should be strengthened to reduce malaria re-infection among ex-patients [13]. Moreover, the study suggested that these activities should be especially focused on improving self-implemented preventive measures among ex-patients traveling to the mountains, and on enhancing knowledge on malaria transmission, especially among indigenous ex-patients.

To accelerate the universal access of malaria patients to diagnostic testing in Palawan, positive health seeking behavior of residents should now be encouraged when malaria infection is suspected. Health seeking behavior for malaria patients has been indicated to be associated with various factors: socio-economic status (age, gender, educational status, financial resources); access to health care providers; perceived severity of disease; and cultural beliefs and practices about the cause and cure of the illness [14–23]. These factors are known to positively or negatively contribute to delays in malaria patients accessing medical facilities, which may lead to death. Few studies have been conducted among Palawan residents to identify factors associated with health seeking behavior.

The objective of the present study was to identify the multi-dimensional factors associated with the appropriate treatment of residents with a history of suspected malaria. The authors hypothesized that their (1) socio-economic status, (2) proximity to a health facility, (3) health seeking behavior (symptoms, treatment, and recovery), (4) knowledge on malaria (symptoms, transmission, vector species, and vector’s most active time), and (5) source of information to raise awareness for malaria prevention would increase the likelihood that residents would receive appropriate treatment.

## Methods

### 2.1. Study site and design

Palawan is an island province lying southwest of the capital island (Luzon). It is mostly covered by tropical rainforests and is one of the most malaria endemic provinces in the country [2–6]. Most infections occur in the tropical rainforest or adjacent areas during the rainy season (June to October). The present study was a cross-sectional study conducted after the rainy season, from January to February in 2012.

In 2010, the province of Palawan was comprised of 367 villages in 23 municipalities with a registered population estimated to be 1,025,800 (male, N = 527,200; female, N = 498,600) [24]. The ethnicity of these residents varied and included Tagalog (the predominant ethnic group in the Philippines), Cuyunon, Hiligaynon, Palawan, Cebuano, Ilocano, and Bisaya.

**Fig 1:**
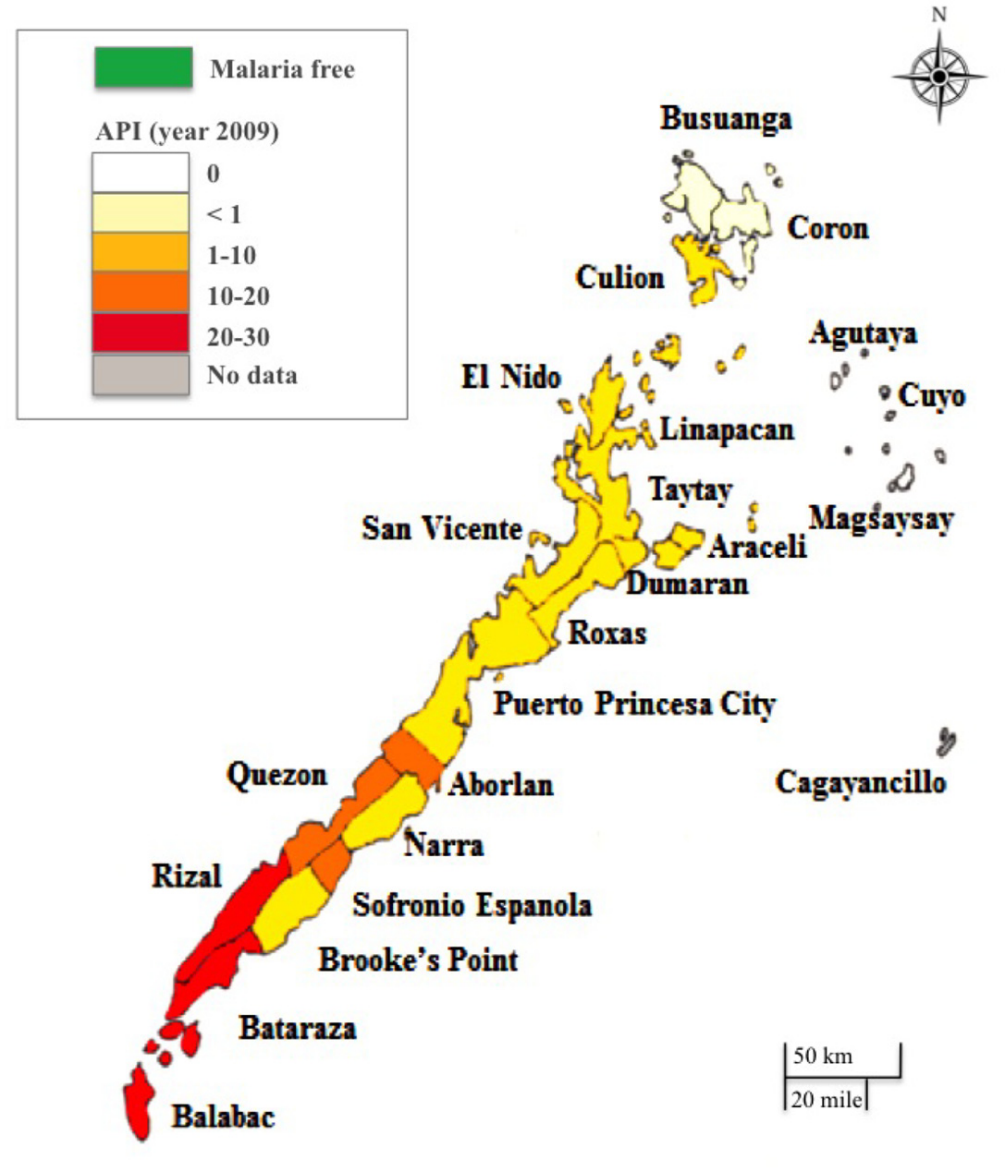
Map of Annual Parasite Incidence (API) (confirmed infections/1,000 population at risk) by municipalities, 2010. This map was constructed by the authors based on the data of provincial health report 2010 [5].

Twenty rural villages were selected for the study from the 137 villages located in 4 endemic municipalities of Palawan: 6 villages in Roxas (northern region), 7 villages in Puerto Princesa City (central region), and 2 and 5 villages in Bataraza and Brooke’s Point, respectively (southern region) (Fig 1). The study villages were distributed throughout the island and were selected with consideration for malaria transmission. In 2011, the APIs of each of the targeted municipalities were: 2.98 in Roxas, 5.87 in Puerto Princesa City, 20.4 in Bataraza, and 9.59 in Brooke’s Point [6]. The remaining high-transmission municipalities (Quezon, Rizal, Sofronio Espanola, and Balabac) were not chosen due to location (mountain or islands) and/or safety concerns (several active militant separatist groups were based around these mountain areas). All of the ex-patients in each of the villages were targeted.

### 2.2. Participants, data collection and ethical considerations

Firstly, permission for the present study was obtained from the governor of the Palawan Provincial Health Office and each respective Municipal Health Office. Lists of malaria patients from 2011 were then collected from each of the rural health units and village health units. These lists were used to select the 20 above-mentioned highly malaria-endemic villages. Data collection, targeting residents with a history of suspected malaria, was carried out in these villages. All households located within one hour from the health center that covers most households in the village were visited. The residents from each household who had most recently been treated for suspected malaria were selected as participants of the present study. Participants who lived near a health center were asked to assemble at the health center; home visits were conducted for those whose homes were more distant. Farmers and gatherers living in distant mountains, migrant agricultural workers, miners, and members of militant separatist groups were excluded from the present study. All 218 participants had a history of illness and were suspected to have been infected with malaria in 2011. Data collection was conducted with the support of microscopists and health center staff.

All participants clearly understood the principles of confidentiality and voluntary participation. Written consent was obtained from all participants before the questionnaires were distributed. No health facility staff members, including microscopists, were involved while the participants were answering the questionnaire. The present study was approved by the Research Ethics Committee of the University of Tokyo (No. 3001) and upheld by the Palawan Provincial Health Office.

### 2.3. Measurements

**Fig 2:**
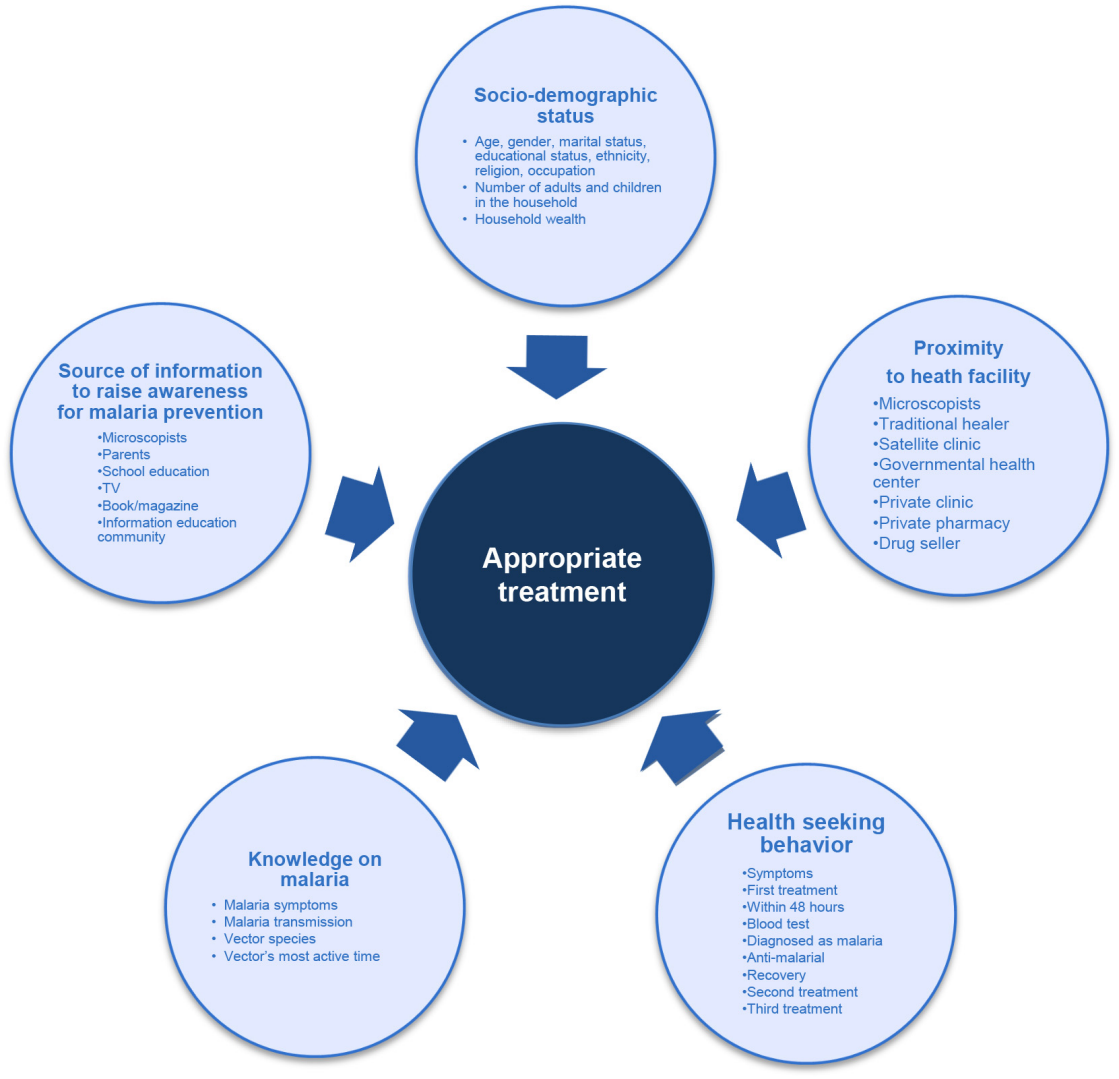
Conceptual framework. The authors hypothesize that an association exists between “appropriate treatment” and the following multi-directional variables: “socio-demographic status,” “proximity to health facility,” knowledge on malaria,” and “participation in community awareness-raising activities for malaria prevention.” Sub-variables of each variable are in the circles of the conceptual framework.

An interviewer-administered structured questionnaire with 65 questions was developed. The questionnaire addressed the following factors: (1) appropriate treatment, (2) socio-demographic status, (3) proximity to health facility, (4) health seeking behavior, (5) knowledge on malaria, and (6) source of information to raise awareness for malaria prevention (Fig 2). The authors also collected data on malaria endemicity from the Provincial Health Office of Palawan.

The questionnaire was developed in English and translated into Tagalog by local malaria experts. Given that some of the participants were members of indigenous groups and did not speak Tagalog, the questionnaire was also translated into indigenous languages. The translators were health center staff members who were fluent in both Tagalog and the target language. The validity and reliability of the whole questionnaire were enhanced with pre-testing and reviewing by two local malaria experts who were fully knowledgeable on the situation of microscopists in Palawan.

#### 2.3.1. Appropriate treatment

Appropriate treatment, the outcome of the present study, was measured based on the participants’ first treatment seeking behavior: (1) participants who received appropriate treatment (microscopists, satellite clinic, government health center, private clinic/practitioner, or NGO clinic or hospital), and (2) participants who did not receive appropriate treatment (private pharmacy, drug seller, or no treatment). Since the two participants who chose a traditional healer (Tagalog: *Albularyo*) as their first treatment seeking action, were very unusual cases, the authors analyzed them separately and did not include them in the SEM model.

#### 2.3.2. Socio-demographic status

Participants were asked 10 questions on socio-demographic status: 6 nominal questions (gender, marital status, educational status, ethnicity, religion, and occupation) and 4 continuous questions (age, number of adults and children in the household, and household wealth). Household wealth was measured by asking participants about the presence of items (electricity, radio, television, refrigerator, bicycle, motorcycle, bike-car, and tin or cement wall) in the household.

#### 2.3.3. Proximity to health facility

Participants were asked if they had any of eight types of medical facility near their house: microscopist, governmental health center, traditional healer, private clinic/ practitioner, NGO clinic or hospital, satellite clinic, private pharmacy, or drug seller.

#### 2.3.4. Health seeking behavior

The following questions were asked to clarify health seeking behavior of the participants: (1) symptoms (stomach ache, fever, diarrhea, nausea, shivering, sweating, or coma); (2) the number of days between the onset of illness and the start of treatment; (3) reason for seeking the first treatment option; (4) treatment received (blood-test, or prescription of anti-malarial); (5) recovery; (6) type of plants and traditional medicine if they were used; and (7) the second and third treatment sought (if sought) and the reason. For the correlation matrix, each “symptom” was counted as “0” for “No” and “1” for “Yes.” The scores were totaled to measure the degree of symptom severity.

#### 2.3.5. Knowledge on malaria

As with the previous studies [12,13], the “knowledge on malaria” questionnaire was derived from the index developed by Yasuoka et al [25]. Quality of participants’ understanding on malaria was measured by 4 parts: 5 questions on “malaria symptoms,” 7 questions on “malaria transmission,” 6 questions on “vector species,” and 4 questions on “vector’s most active time.” Correct answers were coded as “2.” Incorrect answers were coded as “1.” Each of the 4 items was divided by its maximum number of points, to give a maximum score of “1,” which was treated as a continuous variable.

#### 2.3.6. Source of information to raise awareness for malaria prevention

Participants were asked if they had participated in any kind of community awareness-raising activities for malaria prevention. This question was based on 7 sources of awareness-raising activities: microscopists, Information Education Communication, parents, school education, television, or book/magazine.

### 2.4. Statistical analysis

Two types of statistical analysis were conducted after confirming the accuracy of the entered data. Firstly, to provide an overview of the characteristics of the participants, descriptive analysis was conducted. Secondly, to identify the factors associated with appropriate treatment, structural equation modeling (SEM) analysis was used. The correlation of all variables was examined and a path model was built based on the results of bivariate analysis (state model). The fit of the model was examined in terms of degrees of freedom (df), chi-square (CMIN), comparative fit index (CFI), and root mean square error of approximation (RMSEA). According to conventional criteria, a good fit was indicated by CMIN / df < 2, CFI > 0.97, and RMSEA < 0.05, and an acceptable fit by CMIN / df < 3, CFI > 0.95, and RMSEA < 0.08 [26]. All statistical analyses were conducted using SPSS version 18.0 and Amos 18.0 (SPSS Inc., Chicago, IL, USA).

## Results

A total of 218 households participated in the present study: 43 participants (19.7%) from the northern region, 112 from the central region (51.4%), and 63 (28.9%) from the southern region. In each household, the individuals who had the most recent history of illness, and who were suspected to have been infected with malaria, were principally selected to participate in the present study.

### 3.1. Appropriate treatment

**Fig 3:**
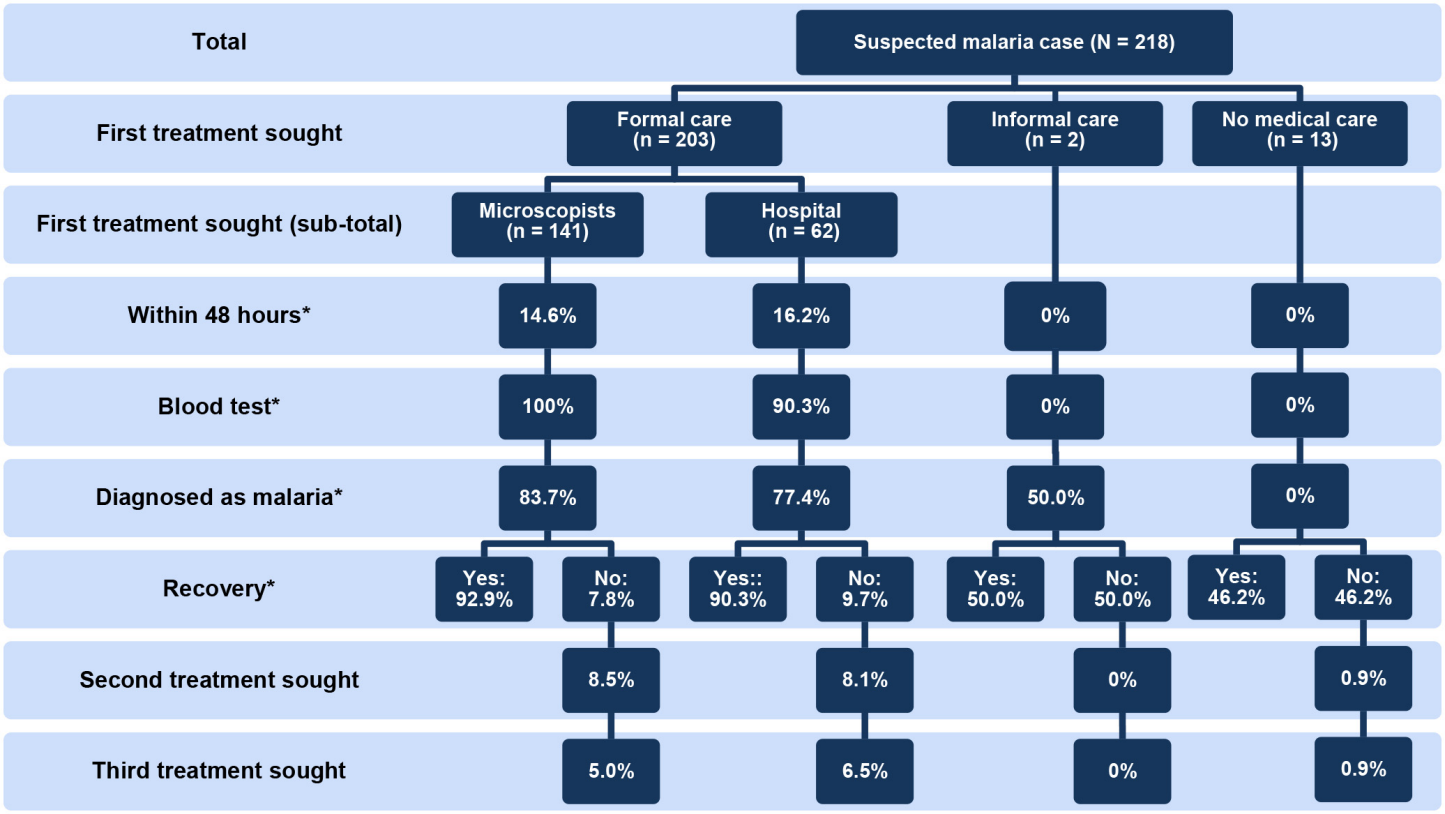
Treatment seeking behavior. * Significant difference (*p* < 0.05) between groups divided by first treatment sought.

Of the 218 selected individuals, 203 (93.1%) were appropriately treated in a formal care setting: 141 (64.7%) by microscopists and 62 (28.4%) in a hospital (Fig 3). Two participants first sought treatment in an informal care setting from a traditional healer. The 13 (6%) remaining respondents did not seek any medical care (no medical care).

### 3.2. Socio-demographic status

Most of the participants were women (80.3%) and were married (87.6%) (Table 1). Nearly half of the participants graduated from high schools or higher education (51.4%). About 70% were Tagalog or an amalgamation of Tagalog, and most were Christian (94.1%) of which more than half were Catholic. As many as 46.3% were homemakers and 20.6% were farmers.

**Table 1:** Nominal socio-economic status variables of participants with respect to first treatment sought.

Socio-economic status	Total (N = 218)	Formal care (N = 203)	Informal care (N = 2)	No medical care (N = 13)	*p*-value^1^
**Gender**					
Male	43 (19.7%)	39 (19.2%)	0 (0%)	4 (30.8%)	0.545
Female	175 (80.3%)	164 (80.9%)	2 (100%)	9 (69.2%)	
**Marital status**					
Never married	15 (6.9%)	14 (6.9%)	0 (0%)	1 (7.7%)	0.107
Married	191(87.6%)	179 (88.1%)	1 (50%)	11 (84.6%)	
Divorced	4 (1.8%)	3 (1.5%)	0 (0%)	1 (7.7%)	
Widowed	8 (3.7%)	7 (3.4%)	1 (50%)	0 (0%)	
**Educational status**					
No grade completed	17 (7.8%)	15 (7.4%)	1 (50%)	1 (7.7%)	0.495
Elementary grade	86 (39.4%)	82 (40.1%)	0 (0%)	4 (30.8%)	
High school	91 (41.7%)	83 (40.9%)	1 (50%)	7 (53.8%)	
College	20 (9.2%)	19 (9.4%)	0 (0%)	1 (7.7%)	
Higher	1 (0.5%)	1 (0.5%)	0 (0%)	0 (0%)	
**Ethnicity**					
Tagalog	62 (28.4%)	58 (28.6%)	0 (0%)	4 (30.8%)	0.840
Tagalog and other ethnics	91 (41.8%)	86 (42.4%)	1 (50%)	4 (30.8%)	
Other	65 (29.8%)	59 (29.1%)	1 (50%)	5 (38.5%)	
**Religion**					
Christian (Catholic)	127 (58.3%)	119 (58.6%)	0 (0%)	8 (61.5%)	0.074
Christian (Non-Catholic)	78 (35.8%)	73 (36.0%)	2 (100%)	3 (23.1%)	
Muslim	6 (2.8%)	4 (2.0%)	0 (0%)	2 (15.4%)	
No religion	7 (3.2%)	7 (3.4%)	0 (0%)	0 (0%)	
**Occupation**					
Homemaker	101(46.3%))	95 (46.8%)	1 (50%)	5 (38.5%)	0.743
Farmer	45 (20.6%)	41 (20.2%)	1 (50%)	3 (23.1%)	
Other	72 (33.0%)	67 (33.0%)	0 (0%)	5 (28.5%)	

^1^Fisher’s exact test between first treatment sought.

The average age of the participants was 39.7 years (SD 13.3). The average number of people in household was 5.4 (SD 2.0), and average number of children per person was 2.9 (SD 1.8). The average household wealth was 1.7 points (SD 1.7) (Table 2).

**Table 2:** Continuous socio-economic status variables of participants with respect to first treatment sought.

Socio-economic status	Total Mean (SD)	Formal care Mean (SD)	Informal care Mean (SD)	No medical care Mean (SD)	ANOVA^1^ (*p*-value)
Age	39.7 (13.3)	39.9 (13.3)	29.0 (14.1)	37.4 (12.5)	0.410
Number of people in household	5.4 (2.0)	5.5 (2.0)	6.0 (2.8)	4.7 (1.8)	0.572
Number of children	2.9 (1.8)	3.0 (1.8)	0.5 (0.7)	2.8 (1.7)	0.245
Household wealth^2^	1.7 (1.7)	1.7 (1.8)	2.0 (2.8)	1.6 (1.6)	0.111

^1^ANOVA between first treatment sought.

^2^This scale scores from 1–8 points, with 1 point each for each of the following: electricity, radio, television, refrigerator, bicycle, motorcycle, bike-car, and tin or cement wall.

### 3.3. Proximity to health facility

Most participants (82.6%) recognized that they were living near a government health center where microscopists were worked (Table 3). However, only half of the participants who received informal care or no medical care (50.0% and 53.8%, respectively) recognized that they were living near a microscopist. Regardless of the participants’ first treatment sought, about half of them recognized that they lived near a traditional healer.

**Table 3:** Proximity to health facility, symptoms, and awareness-raising activities of the participants with respect to first treatment sought (multiple answers allowed).

Variables	Total (N = 218)	Formal care (n = 203)	Informal care (n = 2)	No medical care (n = 13)	*p*-value^1^
Proximity to health facility					
Microscopist	201 (92.2%)	193 (95.1%)	1 (50.0%)	7 (53.8%)	0.001**
Governmental health center	180 (82.6%)	168 (82.8%)	2 (100%)	10 (76.9%)	1
Traditional healer	107 (49.1%)	100 (49.3%)	1 (50.0%)	6 (46.2%)	1
Private clinic/practitioner	92 (42.2%)	85 (41.9%)	1 (50.0%)	6 (46.2%)	0.890
NGO clinic or hospital	84 (38.5%)	78 (38.4%)	1 (50.0%)	5 (38.5%)	0.901
Satellite clinic	76 (34.9%)	72 (35.5%)	1 (50.0%)	3 (23.1%)	0.601
Private pharmacy	31 (14.2%)	26 (12.8%)	1 (50.0%)	4 (30.8%)	0.048*
Drug seller	6 (2.8%)	4 (2.0%)	0 (0%)	2 (15.4%)	0.016**
**Symptoms**					
Stomachache	175 (80.3%)	175 (86.2%)	0 (0%)	0 (0%)	*p* < 0.001***
Fever	172 (78.9%)	172 (84.7%)	0 (0%)	0 (0%)	*p* < 0.001***
Diarrhea	100 (45.9%)	100 (49.3%)	0 (0%)	0 (0%)	*p* < 0.001***
Nausea	95 (43.6%)	95 (46.8%)	0 (0%)	0 (0%)	*p* < 0.001***
Shivering	89 (40.8%)	89 (43.8%)	0 (0%)	0 (0%)	0.019*
Sweating	12 (5.5%)	0 (0%)	2 (100%)	10 (76.9%)	*p* < 0.001***
Coma	3 (1.4%)	3 (1.5%)	0 (0%)	0 (0%)	1
**Source of awareness-raising activities**					
Microscopists	167 (76.6%)	160 (78.8%)	0 (0%)	7 (53.8%)	0.036*
Information Education Communication (IEC)	21 (9.6%)	19 (9.4%)	0 (0%)	2 (15.4%)	0.449
Parents	16 (7.3%)	13 (6.4%)	2 (100%)	1 (7.7%)	0.004**
School education	7 (3.2%)	6 (3.0%)	0 (0%)	1 (7.7%)	0.376
TV	6 (2.8%)	6 (3.0%)	0 (0%)	0 (0%)	1
Book/magazine	6 (2.8%)	5 (2.5%)	0 (0%)	1 (7.7%)	0.332

^1^Fisher’s exact test between first treatment sought.

*Significant difference (0.01 ≤ *p* < 0.05),

**Significant difference (0.001 ≤ *p* < 0.01),

***Significant difference (*p* < 0.001).

### 3.4. Health seeking behavior

Only about 15% of the participants who received formal care (14.6% of the microscopically diagnosed participants and 16.2% of those admitted to hospital) received appropriate treatment within 48 hours (Fig 3). One hundred percent of the participants who visited microscopists and 90.3% of the participants who visited hospital were subjected to blood tests. Malaria was diagnosed in approximately 80% of the participants who visited hospital. Among the participants who visited formal care facilities, 90% recovered. Most of the participants who did not recover received a second and third treatment if needed. Most participants reported that they visited a formal care facility because of the high quality of treatment (80.3%) or because the facility had good equipment (78.9%) (data not shown).

In contrast, none of the participants who received informal care (or those that received no medical care) were treated within 48 hours, nor did they receive a blood test. About half of these patients did not recover but did not seek any further treatment (Fig 3). Among the participants who received no medical care, 3 took plant products and 2 took traditional medicine to treat their symptoms (data not shown). They did not seek any treatment because they thought it was too expensive (15.4%) or because they thought that they would get better soon (15.4%).

Most participants who received formal care reported experiencing stomachache and/or fever (86.2% and 84.7%, respectively), and about half of them reported experiencing diarrhea, nausea, and/or shivering (49.3%, 46.8%, and 43.8%, respectively) (Table 3). None of the participants who went to formal care facilities reported experiencing sweating. Of the patients who received informal care or no medical care, however, most reported experiencing this symptom (100% and 76.9%, respectively). All of the three participants who had severe symptoms (coma) visited formal care facilities.

### 3.5. Knowledge on malaria

“Knowledge on malaria transmission” was significantly higher with participants who received formal care than those who received informal care (*p* = 0.032) (Table 4). In a similar way, other aspects of knowledge (symptoms, and vector species) seem to be higher with participants who received formal care than those who received informal care or no medical care.

**Table 4:** Knowledge on malaria of participants with respect to first treatment sought.

Knowledge on malaria	Total Mean (SD)	Formal care Mean (SD)	Informal careMean (SD)	No medical care Mean (SD)	ANOVA^1^ (*p*-value)
Malaria symptoms	0.59 (0.22)	0.59 (0.23)	0.33 (0.47)	0.45 (0.17)	0.054
Malaria transmission	0.87 (0.25)	0.87 (0.25)^a^	0.42 (0.35)^a^	0.77 (0.34)	0.032*
Vector species	0.33 (0.39)	0.33 (0.40)	0.00 (0.00)	0.26 (0.38)	0.516
Vector’s most active time	0.83 (0.38)	0.82 (0.39)	0.50 (0.71)	0.92 (0.28)	0.398

^1^ANOVA between first treatment sought.

^a^Indicates the combination of two places of first treatment sought that were significantly different by Tukey-Kramer test.

*Significant difference (0.01 ≤ *p* < 0.05)

### 3.6. Source of information to raise awareness for malaria prevention

Among the participants, 78.8% of those who received formal care and 53.8% of those who received no medical care had joined community awareness-raising activities against malaria that had been conducted by microscopists (Table 3). Other sources of information for awareness-raising activities did not attract participants. The two participants who received informal care (traditional healer) had no information on malaria from any source of information other than their parents.

### 3.7. Factors associated with appropriate treatment

To build a hypothetical SEM, bivariate analyses were conducted between all variables. Since the choice of the 2 participants who first sought treatment from a traditional healer, was very unusual, the authors analyzed them separately and did not include them in the SEM model. Several significant correlations were found (Table 5): between “appropriate treatment” and “living near microscopist” (Pearson’s r = 0.40, *p* < 0.01), “not living near drug seller” (Pearson’s r = -0.14, *p* < 0.05), “not living near private pharmacy” (Pearson’s r = -.019, *p* < 0.01), “severe symptoms” (Pearson’s r = 0.21, *p* < 0.01), “knowledge on malaria symptoms” (Pearson’s r = 0.17, *p* < 0.01), and “community awareness-raising activities by microscopists” (Pearson’s r = 0.17, *p* < 0.05). Significant correlations were also found between “living near microscopists” and “not living near private pharmacy” (Pearson’s r = -0.19, *p* < 0.05), and “attending community awareness-raising activities by microscopist” (Pearson’s r = 0.14, *p* < 0.05). People who lived near a drug seller had significantly lower “knowledge on malaria symptoms” (Pearson’s r = -0.16, *p* < 0.05). There was also a significant association between living near a drug seller and not “attending community awareness-raising activities by microscopist” (Pearson’s r = -0.15, *p* < 0.01). Finally, there was a positive correlation between “knowledge on malaria symptoms” and “severe symptoms” (Pearson’s r = 0.37, *p* < 0.01), and “attending community awareness-raising activities by microscopists” (Pearson’s r = 0.38, *p* < 0.01).

**Table 5:** Correlation matrix.

Variables		1	2	3	4	5	6	7
1	Appropriate treatment (1 = no medical care, 2 = formal care)	1						
2	Living near microscopist	0.40**	1					
3	Living near drug seller	-0.14*	-0.04	1				
4	Living near private pharmacy	-0.19**	-0.17*	0.09	1			
5	Severe symptoms	0.21**	0.11	-0.07	0.17*	1		
6	Knowledge on malaria symptoms	0.17*	0.12	-0.16*	0.08	0.37**	1	
7	Community awareness-raising activities by microscopists	0.17*	0.14*	-0.15*	0.31***	0.08	0.38**	1

* Significant difference (0.01 < *p* < 0.05),

** Significant difference (0.001 < *p* < 0.01),

*** Significant difference (*p* < 0.001).

**Fig 4:**
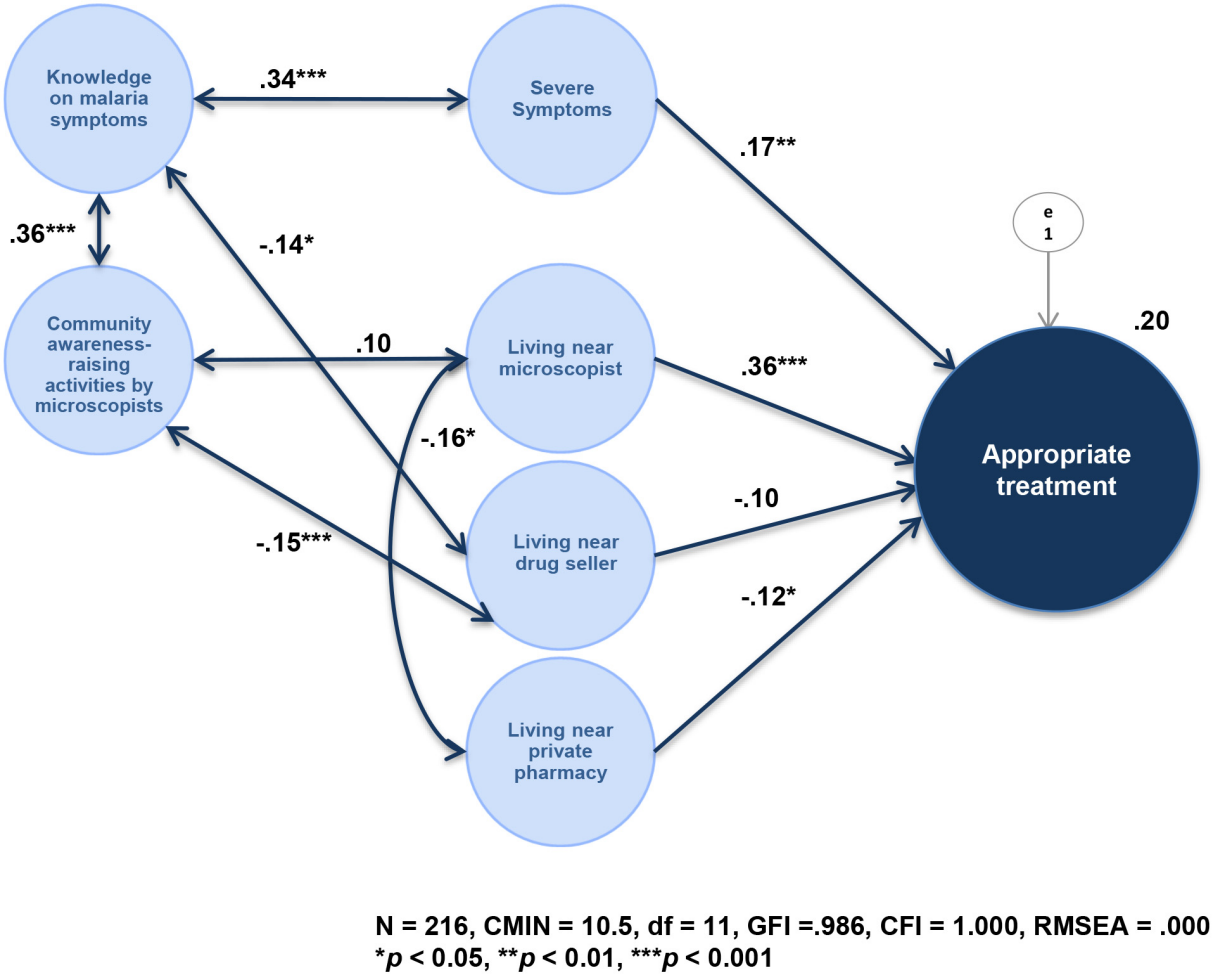
Determinants of appropriate treatment. N = 216, CMIN = 10.5, df = 11, CFI = 1.000, RMSEA = 0.000. * p < 0.05, ** *p* < 0.01, *** *p* < 0.001. Since the two participants who first sought treatment from a traditional healer (Tagalog: Albalaryo) made a very unusual choice, the authors analyzed them separately and did not include them in the SEM model.

Based on these bivariate analyses to examine the relationship between appropriate treatment and other variables, several hypothetical SEMs were built. Fig 4 is the final model that was selected from several models with consideration of the fitness between the data and the model, and of the usability obtained from the results. In this model, the following directional paths were drawn: “severe symptoms” to “appropriate treatment;” “living near microscopist” to “appropriate treatment”; “living near drug seller” to “appropriate treatment;” and “living near private pharmacy” to “appropriate treatment.” Bi-directional paths were drawn as follows: between “knowledge on malaria symptoms” and “severe symptoms,” “community awareness-raising activities by microscopists,” and “living near drug seller”; between “community awareness-raising activities by microscopists” and “living near microscopist,” and “living near drug seller”; between “living near microscopists” and “living near private pharmacy.”

This final model highly fit the data (N = 216, CMIN / df = 0.95, CFI = 1.000, RMSEA = .000). The good fitness of the model was indicated by the following conventional criteria [26]: CMIN / df < 2, CFI > 0.97, and RMSEA < 0.05. In a model using sample size around 200, which is a moderate sample size and is adequate for SEM, those conventional criteria were used to analysis the fitness. CMIN / df, which is a test to assess the fit of a model with consideration of degree of freedom, should be around 0 to 2, and since in the present study CMIN / df was 0.95, it could be said that the fitness was good. Other criteria also indicated good fitness of the model.”Significant associated factors between “appropriate treatment” were “severe symptoms” (path coefficient = 0.17, *p* < 0.01), “living near microscopists” (path coefficient = 0.36, *p* < 0.001), and “not living near private pharmacy” (path coefficient = -0.12, *p* < 0.05). “Knowledge on malaria symptoms” was significantly associated with “severe symptoms” (path coefficient = 0.34, *p* < 0.001), “attending community awareness-raising activities by microscopists” (path coefficient = 0.36, *p* < 0.001), and “not living near drug seller” (path coefficient = -0.14, *p* < 0.05). “Attending community awareness-raising activities by microscopist” was significantly and negatively correlated with “living near drug seller” (path coefficient = -0.15, *p* < 0.001) and “living near microscopists” was significantly and negatively correlated with “not living near private pharmacy” (path coefficient = -0.16, *p* < 0.05). Multiple comparisons were also performed for each pair of groups, with the comparisons denying multicolinearity (Table 5).

## Discussion

The present study was conducted to determine the factors associated with “appropriate treatment” among residents with a history of suspected malaria in the province of Palawan, the Philippines. The final SEM model determined that three factors were independently associated with “appropriate treatment”: “severe symptoms,” “living near microscopist,” and “not living near private pharmacy.”

The microscopists played a significant role in providing access to “appropriate treatment” for all of the participants who had “severe symptoms.” In Palawan, where there is a recognized paucity of formal public and private healthcare providers [1,6], microscopists have brought malaria diagnosis and treatment closer to households. Similarly to the situation in Palawan, CHWs worldwide are used to provide healthcare for a variety of issues when there are critical shortages of highly educated health professionals [27,28]. In serving as healthcare providers, CHWs contribute to facilitating access to malaria diagnosis and treatment, especially in rural communities in South Asia [25], Africa [29,30], and South America [27].

“Severe symptoms” were positively related to having more “knowledge on malaria symptoms.” It was considered that more “knowledge on malaria symptoms” made the participants more aware of their symptoms. Increasing this recognition sensitivity could be a key to accelerating universal access to effective malaria diagnosis and treatment, not only in Palawan but also in the majority of malaria-endemic countries and areas. It is reasonable that people cannot seek appropriate treatment if they lack sufficient knowledge on malaria to realize when they might have the disease. It is known that inappropriate knowledge based on cultural beliefs or practices regarding the cause and cure of the disease significantly hinder proper health seeking behavior [20]. In some African communities it is believed that convulsions (mainly caused by malaria) are related to evil spirits and people who experience convulsions may seek treatment in traditional healers who, they perceive, can combat these spirits [31,32]. Therefore, as an additional strategy, microscopists in Palawan should also focus on community awareness-raising activities to strengthen people’s knowledge on malaria symptoms.

In our previous study [13], three aspects of knowledge (malaria transmission, vector species, and vector’s most active time) were found to be associated with better self-implemented prevention (knowledge on malaria transmission was most highly associated). Knowledge on malaria symptoms, on the other hand, was not associated with better prevention. Interestingly, in the present study we found knowledge on malaria symptoms to be associated with “appropriate treatment,” while the other three knowledge aspects had no impact on “appropriate treatment” seeking behavior. Put simply, we found that knowledge on malaria transmission enhanced preventive measures and that knowledge on malaria symptoms enhanced the seeking of appropriate treatment. Moreover, this important aspect of knowledge, which led people to seek appropriate treatment, was positively and strongly associated with “community awareness-raising activities by microscopists.” Not only were microscopists providing appropriate diagnosis and treatment [12], and educating people to take actions to protect themselves from malaria [12,13], they were also delivering important knowledge, which caused people to seek appropriate treatment. These activities enabled prompt treatment of malaria among residents of Palawan and likely reduced the duration of the disease and saved lives.

It was also important that the residents could recognize that the presence of microscopists near their household gave them free access to appropriate treatment. While all of the participants lived near a health center and were cognizant of this fact, only half of the participants who received informal care or no medical care recognized that they lived near a microscopist. This result suggests that a strategy is needed to make all of the residents aware, for situations in which they suspect that they have been infected with malaria, that they live near a health center in which microscopists are working and that the treatment that the microscopists provide is appropriate and free. It should also be noted that a vulnerability was found in malaria control among ethnic groups [13]. People of the Tagalog ethnicity, took significantly more preventive measures than members of indigenous groups. Therefore, this strategy should be particularly strengthened among indigenous groups, to make them aware of the existence of nearby microscopists.

While it was important for febrile patients to recognize that they lived near a microscopist in order for them to seek appropriate treatment, if they recognized that they lived near a private pharmacy they would be less likely to seek appropriate treatment. In the Philippines, various medicines, including anti-malarial drugs, are available in private pharmacies. While these pharmacies are very important for those who cannot afford healthcare facilities to obtain the minimum amount of drugs to fight their illness, they do not always prescribe appropriate drugs. Now that Palawan has microscopists, who can provide appropriate treatment for free throughout the province, it might be time to conduct targeted training for the private pharmacies in order to increase the provision of appropriate drugs. Residents would be expected to experience a great deal of inconvenience, however, this is important, not only for achieving universal access to proper malaria diagnosis and treatment, but also for reducing the appearance of drug-resistance. At present, in Southeast Asian countries including the Philippines, drug-resistance against all anti-malarial drugs has been reported, even for artemisinin-based combination therapy, which is now the first line therapy for *Plasmodium falciparum* malaria recommended by the WHO [1,33–35]. Moreover, counterfeit anti-malarial drugs are widespread in these areas [36,37]. The significant role of microscopists, rather than private pharmacies, has to be appreciated by the residents.

The present study did not include the participants who received informal care from traditional healers in the final SEM model because of their small number. However, about half of the participants who joined the present study recognized that a traditional healer was located near their household. They have been present from the time the first residents arrived in Palawan and remain there at the time of writing (year 2014). They play a salient role in the healthcare of some community members. Their role in the health systems in Palawan remains to be analyzed.

## Conclusion

In Palawan, where health facilities are limited, microscopists played a significant role in extending treatment access to residents. This enabled all of the study participants with severe symptoms to receive appropriate treatment. On the other hand, knowledge on malaria symptoms was considered to make participants more aware of their symptoms, and improved self-triage. The strengthening of this recognition sensitivity and making residents aware of the presence of nearby microscopists could be the keys to accelerating universal access to effective malaria treatment in Palawan.

## Supporting Information

S1 Dataset Supporting Dataset. (XLSX)

## References

[1] World Health Organization (2013) World malaria report 2013. Available: http://www.who.int/entity/malaria/publications/world_malaria_report_2013/report/en/index.html. Accessed: 2014 Nov 25.

[2] Provincial Health Office of Palawan (1995) Provincial health report, 1995. Palawan: Provincial Health Office of Palawan.

[3] Provincial Health Office of Palawan (2000) Provincial health report, 2000. Palawan: Provincial Health Office of Palawan.

[4] Provincial Health Office of Palawan (2005) Provincial health report, 2005. Palawan: Provincial Health Office of Palawan.

[5] Provincial Health Office of Palawan (2010) Provincial health report, 2010. Palawan: Provincial Health Office of Palawan.

[6] Provincial Health Office of Palawan (2012) Provincial health report, 2012. Palawan: Provincial Health Office of Palawan.

[7] Murray CJ, Rosenfeld LC, Lim SS, Andrews KG, Foreman KJ, Haring D, et al (2012) Global malaria mortality between 1980 and 2010: A systematic analysis. Lancet 379: 413–431. 10.1016/S0140-6736(12)60034-8 22305225

[8] Delacollette C, Van der Stuyft P, Molima K (1996) Using community health workers for malaria control: experience in Zaire. Bull World Health Organ 74: 423–430. 8823965 PMC2486885

[9] Haines A, Sanders D, Lehmann U, Rowe AK, Lawn JE, Jan S, et al (2007) Achieving child survival goals: potential contribution of community health workers. Lancet 369: 2121–2131. 10.1016/S0140-6736(07)60325-0 17586307

[10] Greenwood BM, Bojang K, Whitty CJ, Targett GAT (2005) Malaria. Lancet 365: 1487–1498. 10.1016/S0140-6736(05)66420-3 15850634

[11] Angluben RU, Trudeau MR, Kano S, Tongol-Rivera P (2008) Kilusan Ligtas Malaria: advancing social mobilization towards sustainable malaria control in the province of Palawan, the Philippines. Trop Med Health: 36: 45–49. 10.2149/tmh.36.45

[12] Matsumoto-Takahashi EL, Tongol-Rivera P, Villacorte EA, Angluben RU, Yasuoka J, Kano S, et al (2013) Determining the active role of microscopists in community awareness-raising activities for malaria prevention: a cross-sectional study in Palawan, the Philippines. Malar J 12: 384 10.1186/1475-2875-12-384 24175934 PMC4228447

[13] Matsumoto-Takahashi EL, Tongol-Rivera P, Villacorte EA, Angluben RU, Yasuoka J, Kano S, et al (2014) Determining the impact of community awareness-raising activities on the prevention of malaria transmission in Palawan, the Philippines. Parasitol Int 63: 519–526. 10.1016/j.parint.2014.01.008 24508869

[14] Chibwana AI, Mathanga DP, Chinkhumba J, Campbell CH Jr (2009) Socio-cultural predictors of health-seeking behaviour for febrile under-five children in Mwanza-Neno district, Malawi. Malar J 8: 219 10.1186/1475-2875-8-219 19778433 PMC2763003

[15] Akogun OB, John KK (2005) Illness-related practices for the management of childhood malaria among the Bwatiye people of north-eastern Nigeria. Malar J 4: 13 10.1186/1475-2875-4-13 15723706 PMC553996

[16] Mwangome M, Prentice A, Plugge E, Nweneka C (2010) Determinants of appropriate child health and nutrition practices among women in rural Gambia. J Health Popul Nutr 28: 167–172. 10.3329/jhpn.v28i2.4887 20411680 PMC2980879

[17] Müller O, Traoré C, Becher H, Kouyaté B (2003) Malaria morbidity, treatment-seeking behaviour, and mortality in a cohort of young children in rural Burkina Faso. Trop Med Int Health 8: 290–296. 10.1046/j.1365-3156.2003.01030.x 12667146

[18] Deressa W, Ali A (2009) Malaria-related perceptions and practices of women with children under the age of five years in rural Ethiopia. BMC Public Health 9: 259 10.1186/1471-2458-9-259 19627572 PMC2724516

[19] Sumba PO, Wong SL, Kanzaria HK, Johnson KA, John CC (2008) Malaria treatment-seeking behaviour and recovery from malaria in a highland area of Kenya. Malar J 7: 245 10.1186/1475-2875-7-245 19036154 PMC2607295

[20] Abubakar A, Van Baar A, Fischer R, Bomu G, Gona JK, Newton CR (2013) Socio-cultural determinants of health-seeking behaviour on the Kenyan Coast: A qualitative study. PLoS One 8: e71998 10.1371/journal.pone.0071998 24260094 PMC3832523

[21] Hwang J, Graves PM, Jima D, Reithinger R, Patrick Kachur S (2010) Knowledge of malaria and its association with malaria-related behaviors—Results from the Malaria Indicator Survey, Ethiopia, 2007. PLoS One 5: e11692 10.1371/journal.pone.0011692 20657782 PMC2908133

[22] Feyisetan BJ, Asa S, Ebigbola JA (1997) Mothers’ management of childhood diseases in Yorubaland: the influence of cultural beliefs. Health Transit Rev 7: 221–234. 10176379

[23] Beiersmann C, Sanou A, Wladarsch E, De Allegri M, Kouyaté B, Müller O (2007) Malaria in rural Burkina Faso: local illness concepts, patterns of traditional treatment and influence on health-seeking behaviour. Malar J 6: 106 17686147 10.1186/1475-2875-6-106 PMC1971712

[24] National Statistical Coordination Board (2012) Population of the Philippines census years 1799 to 2010. Manila: National Statistical Coordination Board,.

[25] Yasuoka J, Poudel KC, Poudel-Tandukar K, Nguon C, Ly P, Socheat D, et al (2010) Assessing the quality of service of village malaria workers to strengthen community-based malaria control in Cambodia. Malar J 9: 109 10.1186/1475-2875-9-109 20412600 PMC2873522

[26] Schermelleh-Engel K, Moosbrugger H, Müller H (2003) Evaluating the fit of structural equation models: tests of significance and descriptive goodness-of-fit measures. Methods Psychol Res Online 8: 23–74.

[27] Hazel E, Requejo J, David J, Bryce J (2013) Measuring coverage in MNCH: evaluation of community-based treatment of childhood illnesses through household surveys. PLoS Med 10: e1001384 10.1371/journal.pmed.1001384 23667329 PMC3646213

[28] Satterfield D, Burd C, Valdez L, Hosey G, Shield JE (2002) The “in-between people”: participation of community health representatives in diabetes prevention and care in American Indian and Alaska Native communities. Health Promot Pract 3: 166–175. 10.1177/152483990200300212

[29] Ndiaye Y, Ndiaye JL, Cisse B, Blanas D, Bassene J, Manga IA, et al (2013) Community case management in malaria: review and perspectives after four years of operational experience in Saraya district, south-east Senegal. Malar J 12: 240 10.1186/1475-2875-12-240 23849053 PMC3716525

[30] Mubi M, Janson A, Warsame M, Mårtensson A, Källander K, Petzold MG, et al (2011) Malaria rapid testing by community health workers is effective and safe for targeting malaria treatment: Randomised cross-over trial in Tanzania. PLoS One 6: e19753 10.1371/journal.pone.0019753 21750697 PMC3130036

[31] Makemba AM, Winch PJ, Makame VM, Mehl GL, Premji Z, Minjas JN, et al (1996) Treatment practices for degedege, a locally recognized febrile illness, and implications for strategies to decrease mortality from severe malaria in Bagamoyo District, Tanzania. Trop Med Int Health 1: 305–313. 8673832 10.1111/j.1365-3156.1996.tb00043.x

[32] Ahorlu CK, Dunyo SK, Afari EA, Koram KA, Nkrumah FK (1997) Malaria-related beliefs and behaviour in southern Ghana: implications for treatment, prevention and control. Trop Med Int Health 2: 488–499. 9217705

[33] Mita T, Tanabe K (2012) Evolution of Plasmodium falciparum drug resistance: implications for the development and containment of artemisinin resistance. Jpn J Infect Dis 65: 465–475. 23183197 10.7883/yoken.65.465

[34] Santos G, Torres NV. (2013) New targets for drug discovery against malaria. PLoS One 8: e59968 10.1371/journal.pone.0059968 23555851 PMC3610898

[35] Parija SC, Praharaj I (2011) Drug resistance in malaria. Indian J Med Microbiol 29: 243–248. 10.4103/0255-0857.83906 21860103

[36] Almuzaini T, Choonara I, Sammons H (2013) Substandard and counterfeit medicines: a systematic review of the literature. BMJ Open 3: e002923 10.1136/bmjopen-2013-002923 23955188 PMC3752049

[37] Kelesidis T, Kelesidis I, Rafailidis PI, Falagas ME (2007) Counterfeit or substandard antimicrobial drugs: A review of the scientific evidence. J Antimicrob Chemother 60: 214–236. 10.1093/jac/dkm109 17550892

